# Perceptions of healthcare professionals and people with type 2 diabetes on emotional support: a qualitative study

**DOI:** 10.3399/bjgpopen20X101018

**Published:** 2020-03-18

**Authors:** Michelle Hadjiconstantinou, Alison J Dunkley, Helen Eborall, Noelle Robertson, Kamlesh Khunti, Melanie Davies

**Affiliations:** 1 Research Associate, Diabetes Research Centre, College of Life Sciences, University of Leicester, Leicester, UK; 2 Lecturer, Diabetes Research Centre, College of Life Sciences, University of Leicester, Leicester, UK; 3 Lecturer, Social Science Applied to Healthcare Improvement Research (SAPPHIRE) Group, Department of Health Sciences, University of Leicester, Leicester, UK; 4 Professor, School of Clinical Psychology, University of Leicester, Leicester, UK; 5 Professor of Primary Care Diabetes and Vascular Medicine, Diabetes Research Centre, College of Life Sciences, University of Leicester, Leicester, UK; 6 Professor of Diabetes Medicine and Honorary Consultant, Diabetes Research Centre, College of Life Sciences, University of Leicester, Leicester, UK

**Keywords:** qualitative, type 2 diabetes, healthcare professional training, emotional support, emotional management

## Abstract

**Background:**

Type 2 diabetes mellitus (T2DM) is a demanding condition that impacts the person living with the condition physically and psychologically. Promoting emotional support is a key strategy to improve diabetes care.

**Aim:**

To explore the views and experiences of people with T2DM and healthcare professionals (HCPs) on emotional support in diabetes care, and identify barriers and facilitators to the provision of emotional support in clinical practice.

**Design & setting:**

A qualitative study in England with data collected from four focus groups.

**Method:**

Focus group discussions were conducted with people with T2DM (*n* = 10) and HCPs (*n* = 10). The analysis was informed by the framework method and principles of the constant comparative approach.

**Results:**

Emotional support was lacking in diabetes primary care, and there was a need to normalise the emotional impact of T2DM. Barriers to emotional support included: lack of HCP confidence to discuss emotional issues; lack of counselling training; and time constraints in consultations. Inappropriate use of the word ‘depression’ creates a sense of taboo for those experiencing emotions other than depression.

**Conclusion:**

Consensus between the two target groups indicated a strong need to integrate emotional support in diabetes care, and the need to support and train HCPs in addressing psychosocial aspects of T2DM. Shared language is recommended across diabetes services to appropriately refer to wellbeing. Addressing barriers and considering ways to incorporate emotional management in diabetes consultations is recommended, includings introducing HCP training to increase confidence and enhance counselling skills.

## How this fits in

Despite the recent movement to encourage the provision of emotional support in type 2 diabetes care, this support remains scarce in UK primary care. This article suggests approaches to further enhance the emotional support provided in clinical services, and identifies the following barriers to emotional support: lack of HCP confidence; lack of training; and time restraints. Shared language is needed to appropriately refer to wellbeing.

## Introduction

T2DM is a major global health issue that impacts individuals physically and psychologically. There are approximately 4.8 million people with diabetes in the UK;^1^ 90% of those cases are T2DM.^2^ It has been reported that three in five people with diabetes, including T2DM, experience emotional or mental health problems,^3^ and nearly half the population with T2DM experience day-to-day psychosocial challenges and diabetes-related distress (DRD).^4–6^ In 2008, a UK report identified that 85% of people with T2DM did not receive any psychological support for their condition, which was followed by the publication of a position statement to promote the emotional and psychological support for people with diabetes.^7^ This report, developed by Diabetes UK, aimed to highlight the importance of integrating psychological support in diabetes care on a national level, and aimed to provide recommendations for NHS England, Health Education England, and HCPs for better diabetes care.

Despite initiatives to improve emotional support for individuals with diabetes in the UK, a recent survey of 8596 people from 17 countries (including the UK), indicated that only one-third of people with diabetes are asked by their healthcare team about their psychological wellbeing.^8^ Previous qualitative findings suggest that the availability of emotional support for people with T2DM is limited, and that HCPs lack the skills to address emotional issues in consultations.^9,10^ HCPs may not receive appropriate training in the management of psychological aspects of T2DM, and therefore may be unable to address any issues of wellbeing.^8^

This study aimed to explore the views and experiences of people with T2DM and HCPs regarding emotional support in diabetes, including the identification of barriers and facilitators to the provision of this support in clinical practice. To the authors’ knowledge, this area has not been recently explored in detail in a qualitative setting.

## Method

### Participants and recruitment

The recruitment of participants took a multipronged approach. People with T2DM and HCPs who work in the field of diabetes care were either publicly invited via social media (Facebook and Twitter); directly invited by email, postal invitation, or face-to-face; or responded to a recruitment poster. Additionally, HCPs were approached at local training events and people with T2DM at local ethnic community events via flyers and participant information leaflets.

Participants were purposively sampled to ensure a range in duration of diabetes for people with T2DM, and duration of work in diabetes care for HCPs.

### Topic guide

Topic guides were designed specifically for the present study by two qualitative researchers. Revisions were made in the process of conducting the first focus groups to refine questions and to ensure that the topic guide reflected the research question. Guides included open-ended questions to explore participants’ experiences of living with T2DM; current support available to people with T2DM; and views on emotional support (see Supplementary Box S1). The structure of the topic guide eventually adopted the funnel technique, an approach whereby initial questions are broad and are followed by specific, detailed questions.

### Data collection

Focus groups were conducted at the Leicester Diabetes Centre, England. The focus groups lasted approximately 90 minutes and were facilitated by two experienced qualitative researchers, one with a non-clinical background and one with a clinical background. These were audiorecorded using a digital device with the participants’ consent. The audio files were then transcribed verbatim by an independent, professional transcriber. Initial analyses began during data collection and saturation of data was reached after conducting four focus groups.^11^

### Data analysis

An interpretivist approach was adopted and data were analysed using the framework method^12^ to support thematic analysis. The principles of the constant comparative method were also used (part of the inductive approach of the framework method), where data was managed, mapped, and coded from as many different perspectives as possible.^13^ A thematic framework was identified by the aforementioned two independent researchers and key themes were defined. With the use of NVivo (version 12), the transcripts were coded and labelled. The codes were then charted in a more meaningful and conceptual manner (see Supplementary Box S2). Discrepancies were addressed through discussion between the two coders until agreement was reached.

## Results

Four focus groups were conducted (two with people with T2DM and two with HCPs). The sample comprised 20 individuals, five participants per group (see Supplementary Table S1 for participant characteristics): all ten of the people with T2DM were aged between 40–75 years. The majority of HCPs were aged between 40–59 years (*n* = 7), with the remaining three aged 20–39 years. HCPs’ expertise was diverse, including diabetes specialist nurses (*n* = 6), medical practitioners (*n* = 2), and diabetes specialist dietitians (*n* = 2). Overall, HCP recruitment was not restricted to primary care; however, the majority of HCPs (*n* = 7) were from a primary care background, with the remaining having an integrating role (*n* = 3).

### Themes

For the purpose of this section, people with T2DM will be referred to as ‘PWD’ (person with diabetes). Despite exploring the topic guides in separate groups, the same overall themes emerged from the data: (a) recognition of the emotional impact of T2DM; (b) provision of emotional support in primary care; (c) other sources of emotional support; (d) influences on, and barriers to, emotional support in primary care; and (e) wellbeing versus depression.

### Recognition of the emotional impact of type 2 diabetes

Recognition and normalisation of the emotional impact of T2DM was considered essential to holistic diabetes care. HCPs supported that the emotional challenges of living with a chronic condition must be acknowledged and addressed similarly to other long-term conditions, such as cancer:

*‘I think it’s almost like people need to realise that it’s quite normal to have these emotions.*’

(HCP 1, nurse, female [F])

*‘… it’s very often seen as, ”You’ve only got diabetes, whereas, my friend round the corner has got cancer, or* … *my friend’s got Parkinson’s disease.” It’s “why can’t you cope?” .*.. *It’s a massive issue isn’t it, living with a condition like diabetes on a daily basis.*’

(HCP 1, nurse, F)

Day-to-day emotional challenges appeared to be more acceptable for other long-term conditions, but less so for T2DM. Emotional support seemed to be integrated and accessible in many long-term condition services within and outside the NHS; however, this did not seem to be the case for T2DM:

*‘There’s lots of support for them* [Parkinson’s, stroke]*, be that physical support, but integrated into that is their wellbeing. There’s more of a peer-group support, as well, carer support. Whereas, when you have diabetes, there doesn’t seem to be any support group … apart from Diabetes UK, but that’s not NHS-led.*’

(HCP 3, nurse, F)

### Provision of emotional support in primary care

In comparison to other chronic conditions, the availability and acceptability of emotional support in diabetes was limited. PWD had strong views on the provision of (or lack of) emotional support in primary care at the time of diagnosis. One PWD explicitly shared receiving emotional support from their diabetes nurse:

*‘I got the support from my diabetic nurse right from the word go. But then that put my mind at rest.*’

(PWD 1, F, aged 60–75 years)

However, for most participants this was not the case; many felt they were not provided with the opportunity to have a one-to-one consultation to discuss their concerns and emotions. PWD reported receiving informational support in the form of leaflets instead:

*‘They called me and they said ”right, the results have come back, they’re* [glucose levels] *high, can you come in to the practice and collect your prescription for metformin“ and that was it. The appointment was quick, ”there’s some leaflets here, take whatever you like, off you go”. They* [general practice] *never called back for a review or anything* … ’

(PWD 4, F, aged 40–59 years)

*‘And the diagnosis was “you’re diabetic, here’s some pills, take those, see a nurse every 6–12 months”* … *for the check-ups and the weight, and all that, and it was just a bunch of leaflets as well.*’

(PWD 6, male [M], aged 40–59 years)

These views echoed those of the HCPs focus groups; namely, that standard support provided by primary care is indeed comprised mostly of informational support, with little focus on the emotional demands of T2DM:

*‘I think we’re very good at the very practical things* *…* *so teaching them about targets and numbers, looking at things like driving for instance. I think perhaps we’re not so good at getting into the psyche of the person.*’

(HCP 4, nurse, F)

*‘Yeah I think we’re good at number crunching, and I don’t think we’re good at talking to people about them, and what’s important to them.*’

(HCP 5, nurse, F)

### Other sources of emotional support

For many PWD, their main concern was the uncertainties arising from their condition. All participants agreed that emotional support was important in their diabetes care, but the overriding view was that such support was lacking in primary care. Community leaders were reported to be accessible and helpful sources for emotional support. Two HCPs shared positive experiences on how community support can assist people with T2DM emotionally. They spoke specifically about faith leaders and the role they have within the community to support any concerns or worries about T2DM:

*‘… interestingly, yesterday we ran a group for faith leaders and they were much more in tune to talk about self-esteem, depression, and their emotional side. That’s never come up with HCPs.*’

(HCP 4, nurse, F)

*‘So people use the religious leaders perhaps as a way of support, offloading. I mean presume they’ll be getting direction from them.*’

(HCP 2, nurse, F)

South-Asian populations were able to address emotional issues of T2DM with their communities, and there seemed to be a sense of trust and openness towards faith leaders. It appeared that PWD may attend primary care for ‘practical’ information, and seek emotional support from their community:

*‘So I suppose you could say there’s a cultural side to perhaps who the HCP is, and the patient’s ethnicity, and what they’re able to offer information-wise. It may be they come in* [to primary care] *just for the practical side, and then outside the NHS* … ’

(HCP 3, nurse, F)

To add to this, PWD shared their own experiences of being part of a support group or a physical activity group for people with long-term conditions. These groups, where people are able to talk to others and share personal experiences, can act as a form of peer-support mechanism to PWD. Being able to talk to others in a peer-support setting made them feel confident and relieved:

*‘I’ve got* [a long-term condition]*, so I go to the gym, so at least we can talk to each other there.*’

(PWD 1, F, aged 60–75 years)

*‘You need to talk to some people around you who’ve got similar kind of problems. This is important, because sometimes there’s something bothering you, and they might tell you, “oh, I’ve got this severe itching or something, what do you think it is?” If you just keep it between yourself and the consultant or the doctor, then you’re possibly missing out on this kind of support, which is needed.’*

(PWD 5, male, aged 60–75 years)

### Influences on, and barriers to, emotional support in primary care

Conversations with HCPs frequently referred back to existing limitations in primary care regarding emotional support; three key reasons emerged to explain why emotional support lacks in primary care.

First, lack of time: HCPs and PWD shared similar views that lack of time can be a fundamental barrier to the provision of emotional support. From both groups personal experience, consultations are either very target-driven or time-restricted, which does not allow time to explore and address issues around the emotional management of diabetes:

*‘… I think it’s* [consultation] *very target-driven. Particularly from my perspective in primary care, there’s certain things you need to do within that time.*’

(HCP 4, nurse, F)

*‘GPs have 10 minutes. The nurses have the luxury sometimes of 20, but not always. So time and how you use your consultation is really quite vital to that.*’

(HCP 3, nurse, F)

*‘… for retinal screening, they never have the time to ask you anything. They’re just there to photograph you and say hello and goodbye to you.*’

(PWD 2, M, aged 60–75 years)

*‘They never ask you any questions at all.*’

(PWD 5, M, aged 60–75 years)

Second, lack of confidence: HCPs acknowledged these barriers and agreed that issues must be explored in consultations. However, HCPs expressed fear that such encouragement could ‘*open a can of worms’ *(HCP 2, nurse, F). There was an overall perception that professionals did not feel prepared to provide the appropriate support to address any upcoming emotional issues or concerns, possibly due to lack of confidence:

*‘And “Can I support them? Am I the best person?” It’s having the confidence. Knowing that it’s not our responsibility to sort it out, make them feel better.*’

(HCP 2, nurse, F)

Third, lack of training: HCPs acknowledged that not all HCPs would know how to manage such a consultation with a PWD, suggesting that training in consultation skills is essential. One particular HCP shared their experience on how they approach their consultations, by applying their listening skills:

*‘And sometimes I can just talk to a patient, or sometimes I don’t talk, I just listen. You don’t change any medication, you don’t talk about their results, you just listen* … *so maybe there’s a degree of education, needing staff to look again at how we approach, how you’re able to handle the information you’re given.*’

(HCP 3, nurse, F)

HCP 3 (nurse, F): *‘So motivational interviewing, counselling skills, those key questions that you can ask … ’*

HCP2 (nurse, F): *‘So it’s not being a counsellor, but it’s having those skills.’*

HCP3: *‘… it’s the skills yeah, it’s knowing to address that*.’

(HCP focus group)

For one HCP, it was important not just to be equipped with the right skills but to also provide emotional support at the ‘right time’. For them it was important that a process is in place whereby the PWD receives emotional support at the time of diagnosis:

*‘As a diabetes specialist nurse, it’s maybe 10 years later you’re actually meeting them for the first time, when the diabetes control is way off the scale. So it’s almost, you don’t want to go back and say “well, how are you coping with your diabetes?” because you’re hoping that’s been addressed many years earlier, but probably wasn’t.*’

(HCP 4, nurse, F)

### Wellbeing versus depression

Language and labelling appeared to have a fundamental impact on the behaviour of PWD. Wellbeing may often be referred to as ‘depression’, and for some people this term was perceived as *‘scary’* and *‘daunting’*, reiterating the importance of using appropriate language in consultations and self-management programmes. Participants in both groups believed that emotional support was as important as practical support; however, when emotional wellbeing was constructed as ‘depression’, it elicited a sense of taboo around experiencing emotions other than lowered mood. This ultimately could lead PWD feeling reluctant to disclose their vulnerability due to fears of stigma attached to ‘depression’:

HCP4 (nurse, F): *‘Yeah, because people think ”I’m not depressed”.’*

HCP1 (nurse, F): *‘Even if they are!’*

HCP5 (nurse, F): *‘The word probably scares people* … *if you said emotional support or wellbeing* …’

HCP3 (nurse, F): *‘And I think people don’t see depression the way we see it in its range. They see depression perhaps as somebody very* …’ **

HCP2 (nurse, F): ‘… *who’s on medication.*’

HCP3 (nurse, F): *‘Yeah, very tearful, perhaps very down. And low mood, actually, we know there’s a whole spectrum of depression.*’

(HCP focus group)

HCPs commented that using the term ‘depression’ did not really encompass the emotional impact that PWD actually feel, and that the majority experience other emotional responses, such as DRD or anxiety:

*‘It’s very much a focus on depression, whereas my experience is a lot of people feel very anxious, because the anxiety of thinking “well, am I at increased risk of a heart attack or stroke?”*’

(HCP 1, nurse, F)

Despite depression being perceived as a normal emotional reaction, personal reports from HCPs suggest that PWD could not relate to this emotion or refused to construct their experience with such a negative label.

## Discussion

### Summary

This study explored in detail the experiences and views of people with T2DM and HCPs with regards emotional support in diabetes care. Overall, consensus between HCPs and PWD indicated a strong need for emotional support in T2DM care, and suggested the recognition and normalisation of emotions that accompany the condition. In primary care, diabetes management often centres on managing biometrics, with minimal focus on the provision of emotional support.^14,15^ Despite findings from the DAWN2 study in 2011 demonstrating the importance of psychosocial issues in diabetes care,^8^ the present study suggests that there may still be a significant gap in the provision of emotional support by HCPs.

### Strengths and limitations

This qualitative study was based on the framework method,^12^ which provided a systematic approach to code, manage, and analyse the data in such a way that allows for the unexpected in terms of responses and content. Involving the author as a focus group facilitator may be considered as a limitation, possibly introducing bias to the interpretation of emergent data; however, the involvement of a co-moderator in the focus group discussions, and the facilitator’s experience of conducting focus groups, would have minimised any potential bias.

Initially, the purpose of this study was not to explore emotional support specifically in primary care, but instead to explore emotional support as a whole in diabetes management. The focus on primary care was led by discussions and data from the sample. With newer models of care focusing on the management of diabetes in primary care,^16^ the authors felt the data remained applicable to the management of T2DM, as the majority of adults would be seen in a primary care setting.

The findings add to the literature in regards to the gap in services for T2DM emotional support. For the purpose of the aim of the present study, the authors purposively sampled based on the range in duration of diabetes for people with T2DM and duration of work in diabetes care for HCPs, which led to the collection of rich data. The authors sought to involve a range of professionals who provide direct clinical and emotional care that reflects the multidisciplinary diabetes team; however, the authors recognise that they could have had a greater diversity in professional role if this was included in the sampling strategy. Due to the sample recruited, it was not possible to compare and contrast views within the HCP group. The aim of the study was not to explore the differing roles of HCPs; however, the authors believe this is an important area to consider in future research.

The inclusion of multi-ethnic populations, both in HCPs and PWD focus groups, meant that the topic was explored and scrutinised by two distinct perspectives. Emotional support was also raised in the form of ethnicity-specific support, where individuals reported reaching out to their community groups and faith leaders for reassurance and confidential discussions. As the examples provided in the qualitative study were mainly about South-Asian communities, it would be inappropriate to generalise that all ethnic-specific communities provide such support to their members. Also, due to the small sample size, the group may not necessarily represent the experiences of the overall population with T2DM, such as young adults with T2DM or young adolescents with T2DM. Therefore, future research could also explore the gaps in this particular target group, as well as exploring in detail the importance of religion-based communities.

### Comparison with existing literature

These findings complement results from a recent survey, which aimed to explore the global provision of diabetes self-management education and training for HCPs.^17^ This cross-national survey confirmed that only 19.1% of the 4785 HCPs reported receiving training in psychological management and confirmed that discussions around emotional issues in consultations was limited, with 31–60% of HCPs reporting that this only occurred if initiated by patients.^17^ Two years after that survey, the present study findings confirm that discussions around emotional issues are still avoided, as HCPs are concerned that these might ‘*open a can of worms’*. In addition, the present study findings suggest that emotional support is lacking in primary care due to time constraints in consultations, lack of confidence for HCPs to address the psychosocial aspects of diabetes, and lack of training in psychological management.

These findings suggest that additional training should be provided to HCPs, including motivational interviewing and listening skills, to ensure that emotional support is addressed in consultations. Equipping HCPs with appropriate counselling skills could help build their confidence in addressing psychosocial aspects of diabetes. In addition to providing HCPs with counselling skills, the findings also highlight the importance of providing emotional support at the ‘right time’ and ensuring that PWD receive emotional support at the time of diagnosis onwards.

Furthermore, the findings suggest that the inappropriate use of the word ‘depression’ in clinical practice may create a sense of taboo for PWD who experience emotions other than depression, who as a result may feel pushed away from appropriate care. This highlights the need to distinguish between depression and other emotional states, and to share a common language across services to refer to wellbeing, reiterating the recent report published by NHS England.^18^ The present findings suggest that the term ‘depression’ is commonly used in practices to refer to one’s wellbeing; however, depression does not affect all people with T2DM.^19^ Many people may not relate to this emotional condition, or may be put off being labelled as ‘depressed’. The use of a term that is less clinically-based and more illness-specific, such as ‘wellbeing’ or ‘diabetes-related distress’, may support people to take appropriate actions to address their emotional challenges, and also support HCPs to provide appropriate advice; an approach that was recently suggested in a position statement of the American Diabetes Association.^20^ A mismatch between the terms may magnify misunderstanding between perceived support and provided support. If PWD seek day-to-day support while HCPs diagnose PWD as clinically depressed, such ill-chosen diagnosis may underpin suboptimal management and lead to inappropriate treatment.

### Implications for research and practice

A recently published Diabetes UK handbook,^21^ which acts as a guide to help HCPs support the emotional needs of PWD in consultations, offers guidelines and suggestions to simplify the assessment pathway of psychological problems in diabetes clinical practice. This much needed handbook aims to detect emotional and mental health needs; however, further work is still required to ensure that appropriate counselling skills are practiced by HCPs to complement existing resources. With emotional support becoming a priority in diabetes care, it is essential that HCPs better understand the type of training they require and recognise how best this can be implemented in existing diabetes care guidelines. With evidence showing that training HCPs in psychological skills can have a positive impact on patient care,^10^ further research is required to create appropriate strategies to empower and upskill HCPs in emotional support consultations.

Emotional support is relevant to all aspects of diabetes self-management. Based on the Corbin and Strauss model,^22^ self-management covers three key aspects: medical, behavioural, and emotional. Poor emotional wellbeing can lead to suboptimal self-management, which can essentially influence haemoglobin A1c levels and increase the risk of long-term complications. Therefore, incorporating emotional support within diabetes care ensures that all aspects of diabetes self-management are being addressed to avoid the potential risk or existing complications of suboptimal diabetes management. Future research could explore in more detail how emotional support (or lack of it) may affect people’s self-management of T2DM.

This study explored the perceived needs and perspectives of two distinct target groups; people with T2DM and HCPs. The findings suggest that there is a demand for emotional support in the management of T2DM and a need to upskill HCPs to provide the necessary emotional support to people with T2DM. These findings also suggest the need for shared language across diabetes services that appropriately refers to ‘wellbeing’. It would also be worthwhile considering ways to incorporate emotional management in primary care diabetes consultations and to address barriers to effective person–healthcare communication. This includes introducing HCP training to increase confidence and enhance counselling skills.

## References

[1] Diabetes UK (2020). Number of people with diabetes reaches 4.8 million. https://www.diabetes.org.uk/about_us/news/diabetes-prevalence-2019.

[2] Diabetes UK (2019). Facts & figures. https://www.diabetes.org.uk/Professionals/Position-statements-reports/Statistics.

[3] Diabetes UK (2017). Three in five people with diabetes experience emotional or mental health problems. https://www.diabetes.org.uk/about_us/news/three-in-five-people-with-diabetes-experience-emotional-or-mental-health-problems.

[4] Zanchetta FC, Trevisan DD, Apolinario PP (2016). Clinical and sociodemographic variables associated with diabetes-related distress in patients with type 2 diabetes mellitus. Einstein (Sao Paulo).

[5] Berry E, Lockhart S, Davies M (2015). Diabetes distress: understanding the hidden struggles of living with diabetes and exploring intervention strategies. Postgrad Med J.

[6] Pandit AU, Bailey SC, Curtis LM (2014). Disease-related distress, self-care and clinical outcomes among low-income patients with diabetes. J Epidemiol Community Health.

[7] Diabetes UK (2008). Minding the gap: the provision of psychological support and care for people with diabetes in the UK. https://www.diabetes.org.uk/resources-s3/2017-11/minding_the_gap_psychological_report.pdf.

[8] Nicolucci A, Kovacs Burns K, Holt RIG (2013). Diabetes Attitudes, Wishes and Needs second study (DAWN2™): cross-national benchmarking of diabetes-related psychosocial outcomes for people with diabetes. Diabet Med.

[9] Savage S, Dabkowski S, Dunning T (2009). The education and information needs of young adults with type 2 diabetes: a qualitative study. J Nurs Healthc Chronic Illn.

[10] Graves H, Garrett C, Amiel SA (2016). Psychological skills training to support diabetes self-management: qualitative assessment of nurses' experiences. Prim Care Diabetes.

[11] Rabiee F (2004). Focus-group interview and data analysis. Proc Nutr Soc.

[12] Ritchie J, Spencer L, O'Connor W, Ritchie J, Lewis J (2003). Carrying out qualitative analysis. Qualitative research practice: a guide for social science students and researchers.

[13] Gale NK, Heath G, Cameron E (2013). Using the Framework method for the analysis of qualitative data in multi-disciplinary health research. BMC Med Res Methodol.

[14] Murrels T, Ball J, Maben J (2013). Managing diabetes in primary care: how does the configuration of the workforce affect quality of care?.. https://kclpure.kcl.ac.uk/portal/files/35663953/Managing_diabetes_in_primary_care_Nov_2013.pdf.

[15] Kalra S, Jena BN, Yeravdekar R (2018). Emotional and psychological needs of people with diabetes. Indian J Endocrinol Metab.

[16] Seidu S, Davies MJ, Farooqi A (2017). Integrated primary care: is this the solution to the diabetes epidemic?. Diabet Med.

[17] Byrne JL, Davies MJ, Willaing I (2017). Deficiencies in postgraduate training for healthcare professionals who provide diabetes education and support: results from the Diabetes Attitudes, Wishes and Needs (DAWN2) study. Diabet Med.

[18] NHS England (2018). Language matters: language and diabetes. https://www.england.nhs.uk/wp-content/uploads/2018/06/language-matters.pdf.

[19] Nouwen A (2015). Depression and diabetes distress. Diabet Med.

[20] Young-Hyman D, de Groot M, Hill-Briggs F (2016). Psychosocial care for people with diabetes: a position statement of the American Diabetes Association. Diabetes Care.

[21] Diabetes UK (2019). Diabetes and emotional health — a practical guide for healthcare professionals supporting adults with type 1 and type 2 diabetes. https://www.diabetes.org.uk/professionals/resources/shared-practice/psychological-care/emotional-health-professionals-guide.

[22] Schulman-Green D, Jaser S, Martin F (2012). Processes of self-management in chronic illness. J NursScholarsh.

